# Associations of Commonly Used Concomitant Medications With Survival and Adverse Event Outcomes in Breast Cancer

**DOI:** 10.1002/cam4.71320

**Published:** 2025-10-29

**Authors:** Natansh D. Modi, Ahmad Y. Abuhelwa, Nicole M. Kuderer, Lee X. Li, Gary H. Lyman, Bogda Koczwara, Ganessan Kichenadasse, Luke A. Selth, Ceara Rickard, Mark Haseloff, Agnes Vitry, Richard Woodman, Jessica M. Logan, Huah Shin Ng, Michael D. Wiese, Ross A. McKinnon, Andrew Rowland, Michael J. Sorich, Ashley M. Hopkins

**Affiliations:** ^1^ Flinders University, College of Medicine and Public Health, Flinders Health and Medical Research Institute Adelaide Australia; ^2^ Clinical and Health Sciences, University of South Australia Adelaide Australia; ^3^ Department of Pharmacy Practice and Pharmacotherapeutics, College of Pharmacy University of Sharjah Sharjah UAE; ^4^ Advanced Cancer Research Group Kirkland Washington USA; ^5^ Department of Medicine University of Washington School of Medicine Seattle Washington USA; ^6^ Flinders Centre for Innovation in Cancer, Department of Medical Oncology, Flinders Medical Centre, Flinders University Adelaide Australia; ^7^ Faculty of Health and Medical Sciences, The University of Adelaide Adelaide Australia; ^8^ Consumer Advisory Group, Clinical Cancer Epidemiology Group, College of Medicine and Public Health, Flinders Health and Medical Research Institute, Flinders University Adelaide Australia

**Keywords:** adverse events, breast cancer, cardiovascular drugs, concomitant medications, individual participant data meta‐analysis, proton pump inhibitors, survival outcomes

## Abstract

**Background:**

The impact of commonly used non‐cancer medications on breast cancer outcomes remains underexplored in large datasets.

**Aims:**

To evaluate the associations between commonly used non‐cancer medications and survival as well as adverse events in patients with breast cancer.

**Materials & Methods:**

Individual participant data from 19 breast cancer clinical trials (*n* = 23,211) were pooled. Cox proportional hazards models and logistic regression analyses were used to assess associations between medication use and overall survival, progression‐free survival and grade ≥ 3 adverse events. Analyses were adjusted for demographic, cancer and comorbidity factors.

**Results:**

Proton pump inhibitor use was associated with poorer overall survival (HR 1.19, 95% CI: 1.08–1.30), progression‐free survival (HR 1.11, 95% CI: 1.02–1.21) and an increased risk of grade ≥ 3 adverse events (OR 1.36, 95% CI: 1.21–1.53). Beta‐blockers, ACE inhibitors/ARBs and calcium channel blockers were linked with higher adverse event rates but showed no significant impact on survival. Statins and metformin demonstrated no significant associations with either survival or adverse events.

**Conclusion:**

These findings emphasise the need for careful management of concomitant medications in breast cancer care and support ongoing research to optimise treatment safety and efficacy.

## 1 Introduction

A substantial proportion of patients with breast cancer are multimorbid, with most receiving at least one long‐term medication unrelated to their cancer [1, 2, 3, 4]. Given the high prevalence of co‐existing conditions, such as cardiovascular disease, diabetes and gastro‐oesophageal reflux disease (GORD), concomitant medicines commonly used by patients with breast cancer include antihypertensives, statins, metformin and proton pump inhibitors (PPIs) [2, 3, 5]. However, the association between the use of these non‐cancer concomitant medicines and survival and adverse outcomes in patients with breast cancer has not been extensively evaluated in large, high‐quality datasets.

Emerging evidence suggests that commonly used medicines such as beta‐blockers (BBs), angiotensin‐converting enzyme (ACE) inhibitors/angiotensin II receptor blockers (ARBs), calcium channel blockers (CCBs), metformin, statins and PPIs may impact the therapeutic outcomes of patients with breast cancer [6, 7, 8, 9, 10, 11, 12, 13]. It is proposed that these medicines may influence cancer outcomes through various mechanisms, including altering the pharmacokinetics of anticancer drugs, causing adverse events, impacting the gut microbiota and affecting cancer biology by influencing cellular proliferation, apoptosis and angiogenesis [6, 7, 8, 9, 10, 11, 12, 13]. However, most studies to date have been limited by insufficient data across treatments and breast cancer subtypes and/or an inability to account for potential confounding due to co‐existing comorbidities. Evaluating the impact of the vast number of concomitant medications proposed to have effects on breast cancer outcomes through prospective randomised controlled trials would be impractical due to associated costs, ethical concerns and extended timeframes required to conduct such clinical investigations [14]. Meanwhile, comprehensive access to well‐described clinical and demographic data from clinical trials presents an opportunity to conduct well‐powered detailed investigations.

In this study, we leveraged access to comprehensive and detailed data from clinical trials to perform a pooled individual participant data (IPD) meta‐analysis investigating the associations between commonly used concomitant medicines and the survival and adverse outcomes of patients with breast cancer receiving contemporary anticancer therapies.

## 2 Materials and Methods

### 2.1 Patient Population

This publication is based on research using data from Lilly, Pfizer and Roche that has been made available through Vivli Inc. Vivli has not contributed to or approved and is not in any way responsible for the contents of this publication. Clinical trial IPD was accessed through Vivli to evaluate predictors (such as concomitant medicines) for their ability to identify the likelihood of adverse events and therapeutic outcomes in patients with breast cancer.

This study pools IPD from 19 breast cancer trials, including: CLEOPATRA [NCT00567190, data cut: Feb 2014] [15], EMILIA [NCT00829166, data cut: December 2014] [16], MARIANNE [NCT01120184, data cut: May 2016] [17], TH3RESA [NCT01419197, data cut: Aug 2015] [18], MONARCH1 [NCT02102490, data cut: October 2016] [19], MONARCH2 [NCT02107703, data cut: February 2017] [20], MONARCH3 [NCT02246621, data cut: November 2017] [21], NEXTMONARCH1 [NCT02747004, data cut: June 2020] [22], PALOMA1 [NCT00721409, data cut: November 2013] [23], PALOMA2 [NCT01740427, data cut: February 2016] [24], PALOMA3 [NCT01942135, data cut: October 2015] [25], PHEREXA [NCT01026142, data cut: September 2017] [26], RIBBON1 [NCT00262067, data cut: July 2008] [27], ROSE_TRIO [NCT00703326, data cut: August 2016] [28], APHINITY [NCT01358877, data cut: December 2016] [29], BEATRICE [NCT00528567, data cut: June 2014] [30], HERA [NCT00045032, data cut: June 2015] [31], KATHERINE [NCT01772472, data cut: July 2018] [32] and NEOSPHERE [NCT00545688, data cut: October 2014] [33]. Trials were selected based on the availability of comprehensive IPD on survival, adverse events and concomitant medication use, representing a broad range of breast cancer subtypes and contemporary treatment approaches. Specific eligibility criteria and treatment protocols for each clinical trial are detailed in the cited references and can also be found in their respective entries on ClinicalTrials.gov.

The secondary analysis of anonymised IPD is exempt from review by the local ethics review board (Southern Adelaide Local Health Network Office for Research and Ethics), as it was classified as negligible‐risk research.

### 2.2 Outcomes, Concomitant Medicines and Adjustment Data

The primary outcomes were overall survival (OS) and grade ≥ 3 adverse events (AEs), while progression‐free survival (PFS) and disease‐free survival (DFS) were secondary outcomes. Definitions and criteria for these outcomes, including the versions of NCI CTCAE (National Cancer Institute Common Terminology Criteria for Adverse Events) and RECIST (Response Evaluation Criteria in Solid Tumours) used across the included trials, are summarised in Table S1. Classifications of disease stage (early vs. advanced), hormone receptor and human epidermal growth factor receptor 2 (HER2) status were based on trial‐level definitions as reported in the original datasets. Treatment classifications refer to therapies administered exclusively within the respective trial arms, as per the trial protocols.

Based on prior literature and the pooled cohort's medication use patterns, this study evaluated the association between concomitant non‐cancer medication exposure and outcomes, focusing on medications used by more than 500 participants in the pooled cohort at baseline. In this context, baseline concomitant medication exposure refers to any medication being used at the time of anticancer treatment initiation within the respective clinical trials, regardless of the duration of its pretrial use. According to these criteria, the concomitant non‐cancer medications evaluated in this study included BBs, ACE inhibitors/ARBs, CCBs, statins, metformin and PPIs.

Across all 19 pooled clinical trials, available pre‐treatment characteristic data for model adjustments included concomitant medicine use status, age, body mass index (BMI), oestrogen receptor (ER) status, HER2 status, Eastern Cooperative Oncology Group performance status (ECOG PS), total comorbidity count and comorbidities for which the evaluated concomitant medicines were used.

### 2.3 Statistical Analysis

Associations between concomitant medicine use and OS, DFS and PFS were evaluated using Cox‐proportional hazard analysis. The association between concomitant medicine use and the occurrence of grade ≥ 3 AEs was evaluated using binary logistic regression. Analyses were performed on a per‐protocol basis using complete cases with stratification by study and treatment arm. Associations were reported as hazard ratios (HR) or odds ratios (OR) with 95% confidence intervals (CI). All statistical tests were two‐sided and a *p* value < 0.05 was considered statistically significant. All analyses were adjusted for age, BMI, ER status, performance status, comorbidity count and common comorbidities for which the evaluated concomitant medicines were used. Comorbidities were classified based on definitions provided in the clinical trial datasets.

Forest plots were created to visualise assessed associations, with the R package ‘forestploter’ used for this purpose. Descriptive statistics were used to summarise baseline characteristics. Median follow‐up periods are reported separately for the early‐stage and advanced breast cancer cohorts to reflect the differences in disease trajectory and treatment durations between these groups. Subgroup analyses were conducted for clinically relevant variables, including disease stage (early vs. advanced), presence of liver metastases, breast cancer subtype (e.g., HER2‐positive, HR+/HER2–, triple‐negative) and the type of anticancer therapy.

Between‐subgroup differences in survival and AE outcomes were assessed using the Chi‐square (χ^2^) test of independence. Degrees of freedom (df) were determined as the number of subgroups minus one. *P* values were derived from the Chi‐square statistic to determine whether the observed differences between subgroups were statistically significant. All analyses were conducted using R version 4.1.1.

## 3 Results

### 3.1 Patient Population

This study pooled IPD from 23,211 participants across 19 clinical trials. The median age of the patients was 52 years (Table S2). The cohort's baseline clinical characteristics are presented in Table S2.

The cohort included 13,837 patients (60%) with early‐stage breast cancer and 9374 (40%) with advanced breast cancer. A total of 18,028 patients (78%) had an ECOG PS of 0, while 5096 (22%) had an ECOG PS of ≥ 1. The cohort included 10,348 (45%) patients with a normal BMI, while 4926 (21%), 7214 (31%) and 544 (2%) were classified as obese, overweight, or underweight, respectively. Regarding cancer subtypes, 12,831 (55%) patients had ER‐positive breast cancer, 15,357 (66%) had HER2‐positive breast cancer, 4744 (20%) had hormone receptor‐positive/HER2‐negative breast cancer and 3065 (13%) had triple‐negative breast cancer [TNBC]. The median (range) follow‐up period was 54.9 months for those with early‐stage breast cancer and 41.3 months for those with advanced disease.

In terms of treatment, 13,462 (58%) patients received anti‐HER2 therapies, 2015 (9%) received CDK4/6 inhibitors, 2592 (11%) received hormonal therapy, 2088 (9%) received chemotherapy alone and 2127 (9%) received VEGF therapy.

A total of 6319 (27%) patients had a history of comorbidities typically managed with BBs, ACE inhibitors/ARBs, or CCBs—including 5628 (24%) patients who had a history of hypertension. Additionally, 2006 (9%) patients had conditions commonly treated with PPIs, including 1253 (5%) patients with a history of GORD or dyspepsia. A history of dyslipidaemia, often treated with statins, was reported in 2263 (10%) patients and 1354 (6%) patients had a history of diabetes commonly treated with metformin. The median number of comorbidities per patient was 2 [1, 2, 3, 4].

At baseline, 1797 (8%) patients were using BBs, 2894 (12%) were on ACE inhibitors/ARBs, 1533 (7%) were taking statins, 1193 (5%) were on CCBs, 731 (3%) were using metformin and 1881 (8%) were on PPIs.

### 3.2 Proton Pump Inhibitors

**FIGURE 1:**
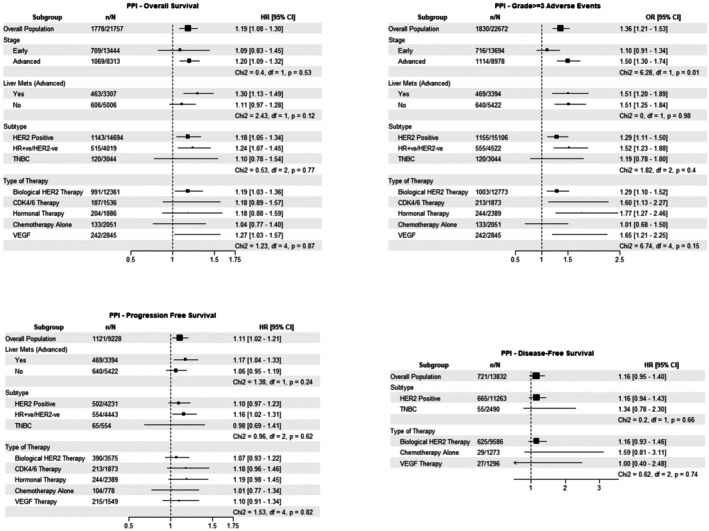
PPI Forest Plot.

The associations between PPI use and OS, PFS, DFS and grade ≥ 3 AEs are demonstrated in Figure 1. In the overall cohort, PPI use was significantly associated with poorer OS (HR 1.19, 95% CI: 1.08–1.30) and PFS (HR 1.11, 95% CI: 1.02–1.21) and an increased occurrence of grade ≥ 3 AEs (OR 1.36, 95% CI: 1.21–1.53). No significant association between PPI use and DFS was identified. There was no significant difference in the observed PPI associations between subgroups based on stage, presence of liver metastases, treatment type, or breast cancer subtype for OS and PFS. Despite adjusting for age, BMI, ECOG PS, ER status, as well as the number of comorbidities and the common comorbidities for which PPIs are used, patients with advanced‐stage disease had a significantly higher risk of grade ≥ 3 AEs with PPI use than patients with early‐stage disease (χ^2^ = 6.28, df = 1, *p* = 0.01).

### 3.3 Beta‐Blockers

**FIGURE 2:**
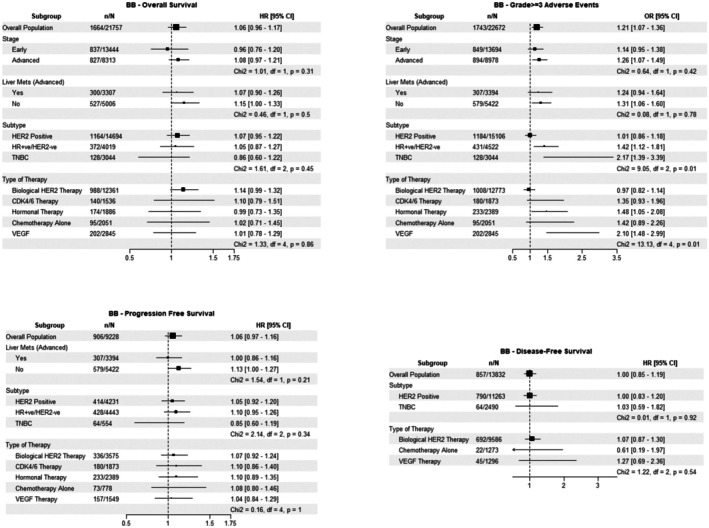
Beta‐blocker Forest Plot.

In the pooled cohort, BB use was significantly associated with a higher risk of developing grade ≥ 3 AEs (OR 1.21, 95% CI: 1.07–1.36) (Figure 2). No significant association between BB use and OS, PFS, or DFS was identified. For grade ≥ 3 AEs, there were no differences in the identified BB associations between subgroups based on disease stage or the presence of liver metastases. However, among BB users, the risk of grade ≥ 3 AEs was higher for patients with HR‐positive/HER2‐negative disease and TNBC compared to patients with HER2‐positive disease (χ^2^ = 9.05, df = 2, *p* = 0.01). Additionally, the risk of grade ≥ 3 AEs was higher for patients receiving hormonal and VEGF therapies compared to those treated with anti‐HER2 medicines, CDK4/6 inhibitors, or chemotherapy alone (χ^2^ = 13.13, df = 4, *p* = 0.01).

### 3.4 ACE Inhibitors/ARBs

**FIGURE 3:**
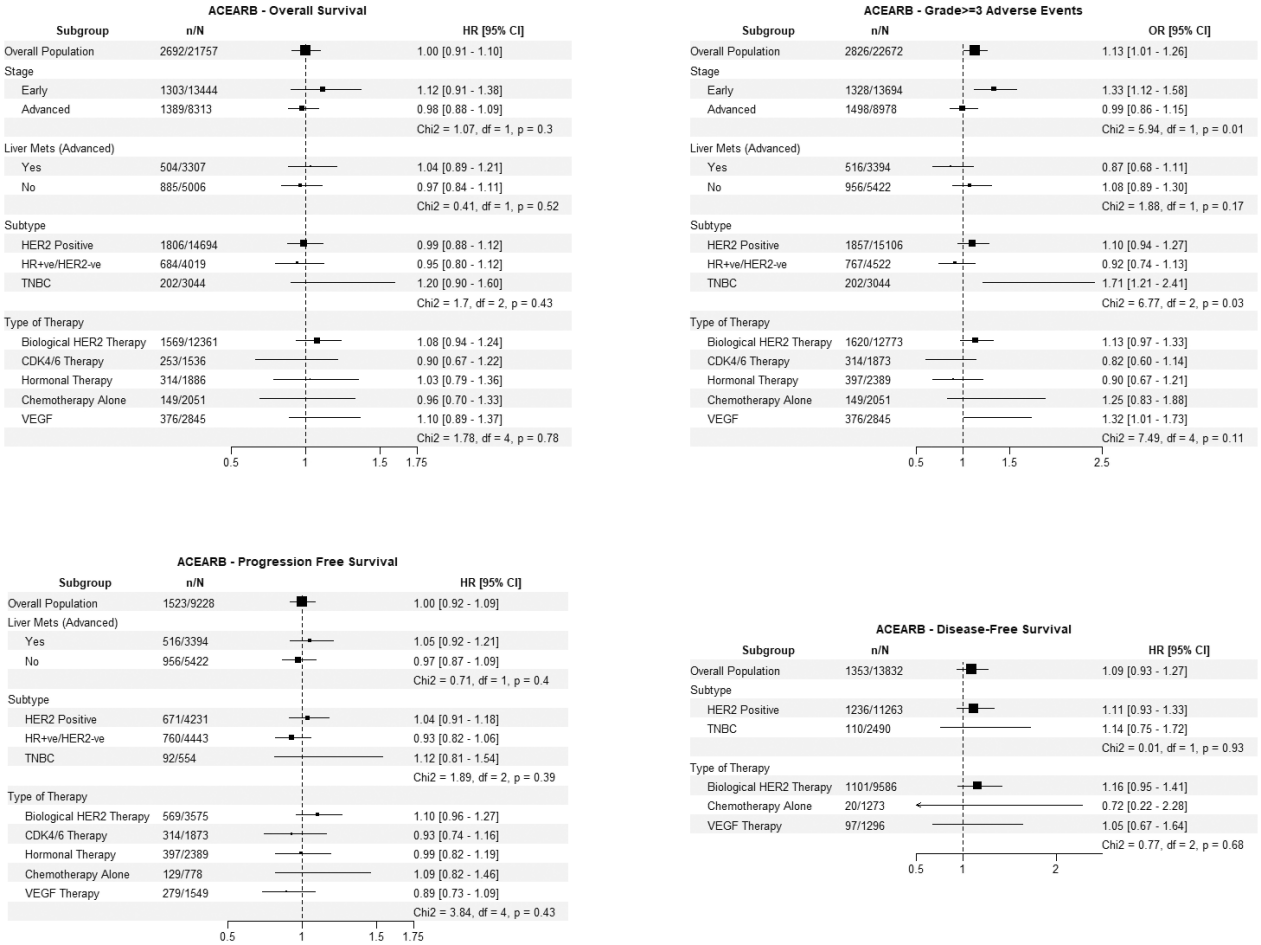
ACEARB Forest Plot.

In the pooled cohort, ACE inhibitor/ARB use was significantly associated with a higher risk of developing grade ≥ 3 AEs (OR 1.13, 95% CI: 1.01–1.26), (Figure 3). However, there was no association between ACE inhibitor/ARB use and OS, PFS, or DFS. For grade ≥ 3 AEs outcomes, there were no differences in the identified ACE inhibitor/ARB associations based on the presence of liver metastases or cancer treatment. However, the risk of grade ≥ 3 AEs in ACE inhibitor/ARB was higher for patients with early‐stage disease compared to advanced‐stage disease (χ^2^ = 5.94, df = 1, *p* = 0.01). The risk of grade ≥ 3 AEs was also higher in patients with TNBC than in patients with HER2‐positive and HR‐positive/HER2‐negative disease (χ^2^ = 6.77, df = 2, *p* = 0.03).

### 3.5 Calcium Channel Blockers

**FIGURE 4:**
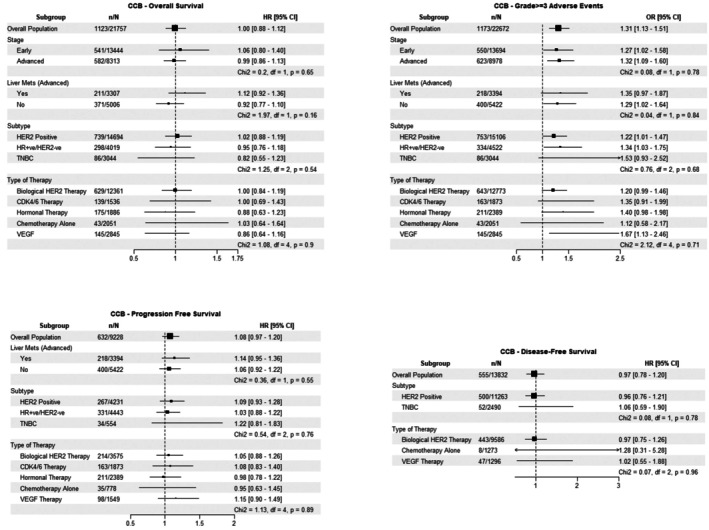
CCB Forest plot.

In the pooled cohort, CCB use was significantly associated with a higher risk of developing grade ≥ 3 AEs (OR 1.31, 95% CI: 1.13–1.51) (Figure 4). No significant association between CCB use and OS, PFS, or DFS was identified. Additionally, no significant differences in the identified association between CCB use and the occurrence of grade ≥ 3 AEs were observed between subgroups based on disease stage or subtype, presence of liver metastases or cancer treatment.

### 3.6 Statins and Metformin

In the pooled cohort, there were no associations between statin or metformin use with OS, PFS, DFS and grade ≥ 3 AEs (Figures S1 and S2).

## 4 Discussion

Defining the impact of concomitant medicines on breast cancer outcomes is essential for guiding treatment decisions and improving patient care, particularly as advancements in therapy are extending survivorship after diagnosis. This study provides the most comprehensive evaluation to date of the impact of commonly used concomitant medicines on breast cancer outcomes, drawing on data from over 23,000 patients across 19 clinical trials. Concomitant PPI use was significantly associated with poorer OS, PFS and an increased occurrence of grade ≥ 3 AEs. Furthermore, a higher risk of developing grade ≥ 3 AEs was observed in patients who used BBs, ACE inhibitors/ARBs and CCBs.

The association between PPIs and poorer outcomes in this study aligns with a growing body of evidence suggesting potential risks associated with their use in patients with cancer [34, 35, 36, 37, 38]. These findings are particularly concerning given the widespread overprescribing of PPIs in oncology care [39]. One plausible mechanism underlying the association between PPI use and worse outcomes in cancer patients relates to potential direct and comorbid disease‐related disruptions of the gut microbiota–immune system axis [40]. The gut microbiome is increasingly recognised for its role in regulating systemic immune responses, which are important for anti‐tumour activity [41]. Additionally, PPIs may alter the pharmacokinetics of anticancer therapies, including CDK4/6 inhibitors, chemotherapy agents and immune checkpoint inhibitors [12, 42], potentially affecting their efficacy. Our findings emphasise the careful and judicious use of PPIs in patients with cancer, particularly those with breast cancer and highlight the necessity for ongoing research into their safety.

In our analysis, no significant associations were found between the use of BBs, ACE inhibitors/ARBs, or CCBs with survival outcomes in this large, diverse cohort. However, an increased risk of grade ≥ 3 AEs was observed. Previous studies on the influence of these medicines on breast cancer outcomes have provided conflicting evidence. For instance, BBs have been reported to inhibit tumour proliferation and migration by blocking adrenergic signalling [43, 44], supporting findings from several small studies showing improved survival outcomes [7, 44]. However, other studies demonstrated either worse outcomes or no associations between BB use and breast cancer outcomes [6, 45]. Whilst ACE inhibitors and ARBs have been proposed to affect breast cancer through their impact on the renin‐angiotensin system [46], meta‐analyses across smaller studies are inconsistent [9]. CCBs have also been implicated in both promoting cancer progression through inhibition of apoptosis and possibly suppressing tumour progression via immunosuppressive effects [47, 48]. Our own findings using a much larger dataset are therefore important in providing reassurance that BBs, ACE inhibitors/ARBs and CCBs do not worsen survival outcomes in patients with breast cancer and support their use when necessary. However, the increased risk of adverse events, potentially linked to cardiovascular, renal, or frailty mechanisms, also highlights the need for careful monitoring of patients taking these drugs, particularly those with additional risk factors for treatment‐related adverse events. No statistically significant interactions between PPI use and comorbidity count were identified for OS, PFS, DFS and grade ≥ 3 AEs.

This study found no significant associations between statin or metformin use and therapeutic outcomes in breast cancer, providing reassurance about their safety. These findings contrast with previous studies that suggested potential benefits, particularly for metformin, which has been linked to improved cancer outcomes [49, 50, 51, 52]. Similarly, statins, known for their cholesterol‐lowering properties, have been hypothesised to offer anticancer benefits through mechanisms such as modulating cellular proliferation and inflammation [53, 54, 55]. The absence of significant effects in our pooled cohort may suggest that any potential anticancer beneficial effects from either statins or metformin are more modest than previously thought or could be limited to specific subpopulations not adequately represented in this analysis.

Clinical trials, particularly RCTs, are the backbone of evidence‐based medicine; however, their strict inclusion criteria can limit the generalisability of results to broader real‐world practice [56]. Our study utilises data from 19 high‐quality clinical trials involving over 23,000 patients, thereby capturing a wide array of breast cancer subtypes and treatments. While this increases the scope of our analysis, we acknowledge that the dataset remains subject to selection bias inherent in clinical trial populations. Nevertheless, the large sample size of our dataset enhances statistical power and enables the detection of small but clinically important effects. The well‐described nature of our data, as is often only available in rigorously collected clinical trial data, also allowed us to adjust for multiple potential confounders, including age, BMI, oestrogen receptor status, performance status and comorbidity burden. Moreover, the higher‐than‐typical prevalence of HER2‐positive breast cancer in our study population may limit the broader generalisability of our findings [57]. Additional limitations to the study include that as a posthoc observational study, we cannot establish definitive causal relationships between concomitant medicine use and therapeutic outcomes [58]. Residual confounding could also persist despite adjustment for known variables, particularly given the complexity of comorbid conditions and how they interact with each other and variations in treatment regimens. Finally, the lack of detailed data on dosing, duration, polypharmacy and adherence to concomitant medicines limits our ability to fully assess their impact on outcomes.

Despite these limitations, our findings represent a significant step forward in understanding the interplay between commonly used concomitant medicines and breast cancer outcomes. To build on these findings, future studies should aim to confirm these associations through prospective studies and explore the underlying mechanisms, including the potential impact of PPIs on gut microbiome dysregulation or their role as proxies for steroid use (since PPIs are commonly prescribed to prevent steroid‐induced gastrointestinal irritation) [59]. Comprehensive analyses of non‐haematologic toxicities, including the differences in adverse events associated with medications like PPIs versus antihypertensives (e.g., expected cardiovascular‐type AEs from antihypertensives), would provide clinicians with better guidance on monitoring and managing associated risks. Integrating key prognostic factors, such as tumour stage, number of positive lymph nodes and tumour grade, will strengthen future analyses. Furthermore, studies should aim to standardise the reporting of medication use in clinical trials to enable detailed polypharmacy analyses. Our study also highlights the importance of ongoing comprehensive clinical trial data access. As cancer therapies advance rapidly, timely and frequent sharing of new trial data is important to sustain the relevance of analyses and effectively guide evidence‐based patient care [60].

In conclusion, our study identifies a significant association between PPI use and poorer OS and PFS in patients with breast cancer, along with a higher risk of grade ≥ 3 adverse events, challenging their assumed safety in breast cancer care. Moreover, the increased risk of grade ≥ 3 adverse events associated with BBs, ACE/ARBs and CCBs warrants increased monitoring of patients using these drugs. Although our analysis does not establish direct mechanistic links, it raises important questions about routinely used concomitant medicines and the need for more personalised approaches to care that balance their potential risks and benefits.

## Author Contributions

All authors contributed to the study design, data analysis, data interpretation and drafting of the manuscript. All authors have read and approved the final version of the manuscript. The corresponding author attests that all listed authors meet authorship criteria and that no others meeting the criteria have been omitted.

## Disclosure

During the preparation of this work, the authors used ChatGPT and Grammarly AI to assist in the formatting and editing of the manuscript to improve the language and readability. After using these tools, the authors reviewed and edited the content as needed and took full responsibility for the content of the publication.

## Ethics Statement

Secondary analysis of anonymised IPD was exempted from review by the Southern Adelaide Local Health Network, Office for Research and Ethics, as it was classified as minimal risk research.

## Conflicts of Interest

A.R. and M.J.S. Sorich are recipients of investigator‐initiated funding for research outside the scope of the current study from AstraZeneca, Boehringer Ingelheim, Pfizer and Takeda. A.R. is a recipient of speaker fees from Boehringer Ingelheim and Genentech outside the scope of the current study. A.M.H. is a recipient of investigator‐initiated funding for research outside the scope of the current study from Boehringer Ingelheim. N.M.K. reports receiving consulting fees from AstraZeneca, Janssen, Pfizer Inc., Bristol Myers Squibb, BeyondSpring Inc., G1 Therapeutics Inc., Sandoz, Seagen Inc. and Fresenius Kabi outside the submitted work. GH Lyman reported receiving consulting fees from AstraZeneca, Sandoz, G1 Therapeutics Inc., BeyondSpring Inc. and Fresenius Kabi outside the submitted work. The author team has no other support, financial relationship, or other relationship activities that could appear to have influenced the submitted work.

## Supporting information


**Table S1:** Outcome definitions and criteria across included studies.
**Table S2:** Demographics.
**Figure S1:** Statin forest plot.
**Figure S2:** Metformin forest plot.

## Data Availability

This publication is based on research using data from Lilly, Pfizer and Roche that has been made available through Vivli Inc. Data can be searched via the data IDs provided in the methods, but a request must be logged in order to access the data. Vivli has not contributed to or approved and is not in any way responsible for the contents of this publication. The code for data processing and visualisation is written in R and available upon request (N.M.: natansh.modi@flinders.edu.au).
