# Supplementary material for: Associations of Commonly Used Concomitant Medications With Survival and Adverse Event Outcomes in Breast Cancer
Source: Cancer Med. 2025 Oct 29;14(21):e71320. doi: 10.1002/cam4.71320 (PMC12571984; doi:10.1002/cam4.71320)
Supplement: Supplementary file 1 — Table S1: Outcome definitions and criteria across included studies. Table S2: Demographics. Figure S1: Statin forest plot. Figure S2: Metformin forest plot. [file CAM4-14-e71320-s001.docx]

**Supplementary Tables and Figures**

Supplementary Table 1: Outcome Definitions and Criteria Across Included Studies

| **Study** | **OS** | **PFS** | **DFS** | **AEs** |
| --- | --- | --- | --- | --- |
| BEATRICE | Randomisation to last follow-up or death | Not assessed | Time to ipsilateral invasive breast tumour recurrence, local-regional recurrence, distant recurrence, death, or secondary cancer | NCI CTCAE v3.0 |
| NEOSPHERE | Not assessed | Investigator-defined using RECIST 1.0 | Time from surgery (disease-free) to progressive disease or death | NCI CTCAE v3.0 |
| CLEOPATRA | Randomisation to last follow-up or death | Investigator-defined using RECIST 1.0 | Not assessed | NCI CTCAE v3.0 |
| EMILIA | Randomisation to last follow-up or death | Independently assessed using RECIST 1.0 | Not assessed | NCI CTCAE v3.0 |
| PALOMA1 | Randomisation to last follow-up or death | Investigator-defined using RECIST 1.0 | Not assessed | NCI CTCAE v3.0 |
| PHEREXA | Randomisation to last follow-up or death | Investigator-defined using RECIST 1.0 | Not assessed | NCI CTCAE v3.0 |
| RIBBON1 | Randomisation to last follow-up or death | Investigator-defined using RECIST 1.0 | Not assessed | NCI CTCAE v3.0 |
| ROSE_TRIO | Randomisation to last follow-up or death | Investigator-defined using RECIST 1.0 | Not assessed | NCI CTCAE v3.0 |
| APHINITY | Randomisation to last follow-up or death | Not assessed | Time to ipsilateral invasive breast tumour recurrence, local-regional recurrence, distant recurrence, death, or secondary cancer | NCI CTCAE v4.0 |
| KATHERINE | Randomisation to last follow-up or death | Not assessed | Time to ipsilateral invasive breast tumour recurrence, local-regional recurrence, distant recurrence, death, or secondary cancer | NCI CTCAE v4.0 |
| TH3RESA | Randomisation to last follow-up or death | Investigator-defined using RECIST 1.1 | Not assessed | NCI CTCAE v4.0 |
| MARIANNE | Randomisation to last follow-up or death | Independently assessed using RECIST 1.1 | Not assessed | NCI CTCAE v4.0 |
| MONARCH 1-3 | Randomisation to last follow-up or death | Investigator-defined using RECIST 1.1 | Not assessed | NCI CTCAE v4.0 |
| NEXTMONARCH1 | Randomisation to last follow-up or death | Investigator-defined using RECIST 1.1 | Not assessed | NCI CTCAE v4.0 |
| PALOMA 2 & 3 | Randomisation to last follow-up or death | Investigator-defined using RECIST 1.1 | Not assessed | NCI CTCAE v4.0 |
| HERA | Randomisation to last follow-up or death | Not assessed | Time to loco-regional/distant recurrence, contralateral cancer, second malignancy, or death | NCI CTCAE v2.0 |

**Supplementary Table 2: Demographics**

| Clinical Characteristics | **Total No. (23,211)** |
| --- | --- |
| **Age (years)** |  |
| Median (IQR) | 52 (44 - 60) |
| Missing | 2 (<1%) |
| **BMI - WHO classification** |  |
| Normal | 10,348 (45%) |
| Obese | 4,926 (21%) |
| Overweight | 7,214 (31%) |
| Underweight | 544 (2%) |
| Missing | 179 (1%) |
| **ECOG PS** |  |
| 0 | 18,028 (78%) |
| ≥ 1 | 5,096 (22%) |
| Missing | 87 (<1%) |
| **Estrogen Receptor Status** |  |
| Yes | 12,831 (55%) |
| No | 10,096 (43%) |
| Missing | 284 (1%) |
| **Comorbidity Count** | 2 (1-4) |
| **Breast Cancer Subtype** |  |
| HER2-Positive | 4,744 (20%) |
| HR-Positive/HER2-Negative | 15,357 (66%) |
| Triple Negative | 3,065 (13%) |
| Missing | 45 (<1%) |
| **STAGE** |  |
| Early | 13,837 (60%) |
| Advanced | 9,374 (40%) |
| **Type of Therapy** |  |
| Anti-HER2 | 13,462 (58%) |
| CDK4/6 | 2,105 (9%) |
| Hormonal | 2,592 (11%) |
| Chemotherapy Alone | 2,088 (9%) |
| VEGF | 2,127 (9%) |
| **Concomitant Medicines Users** |  |
| Beta-Blocker | 1,797 (8%) |
| ACE Inhibitor/ARB | 2,894 (12%) |
| Statin | 1,533 (7%) |
| Calcium Channel Blocker | 1,193 (5%) |
| Metformin | 731 (3%) |
| Proton-pump Inhibitor | 1,881 (8%) |
| Data are median (IQR) or number of patients (%)  VEGF = Vascular Endothelial Growth Factor  CDK = Cyclin Dependent Kinase | |

**Supplementary Figure 1: Statin Forest Plot**


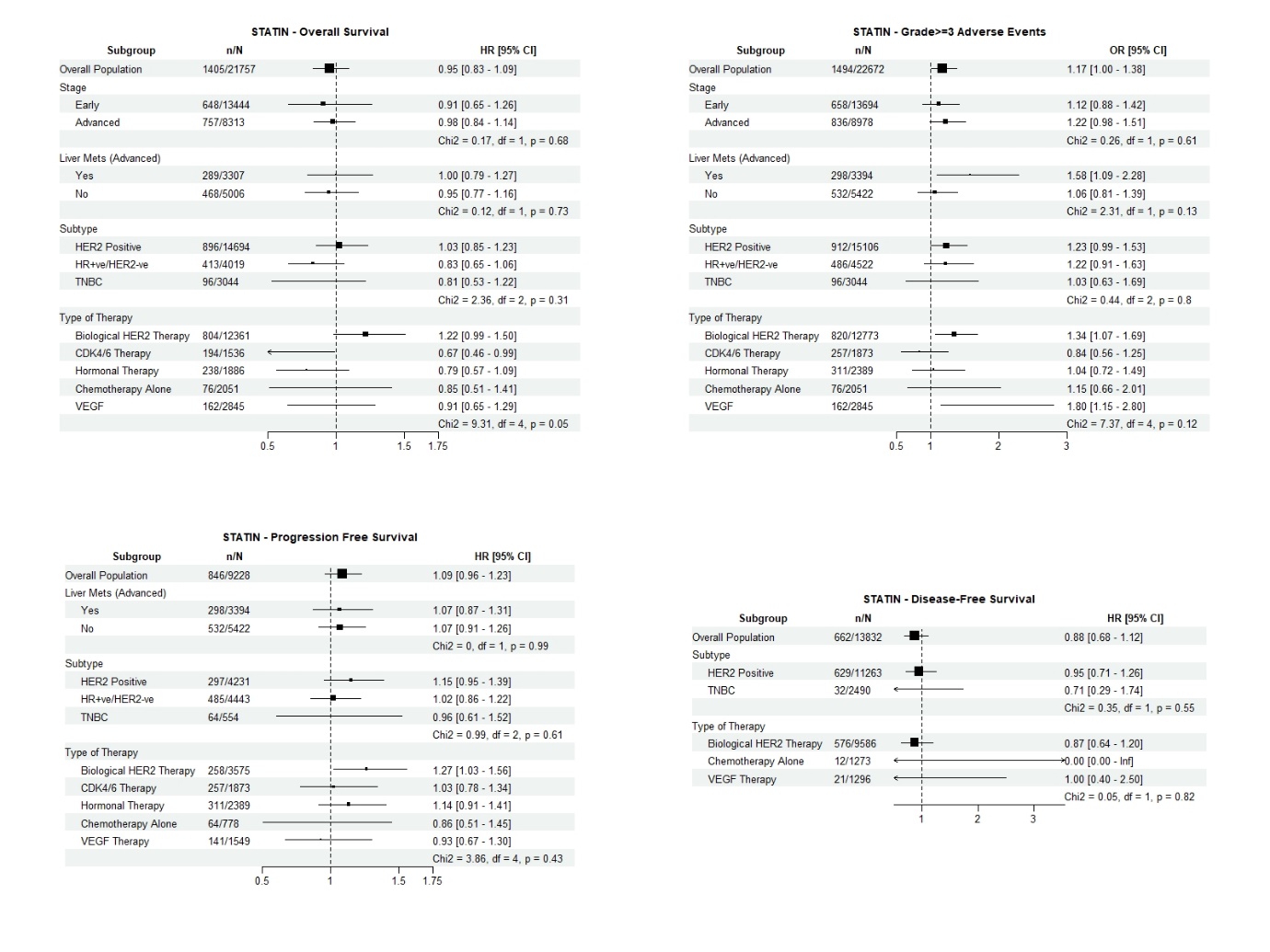


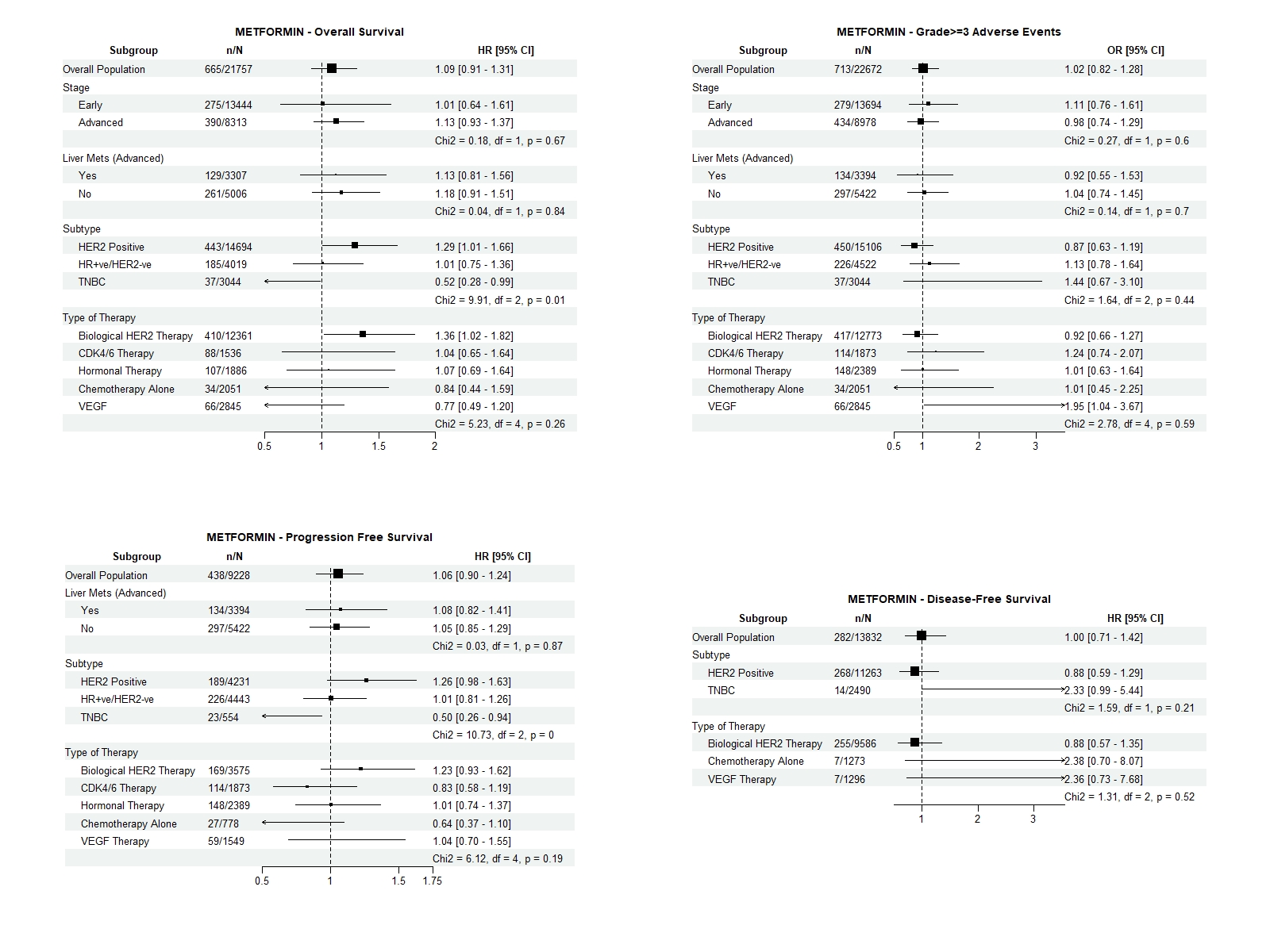
**Supplementary Figure 2: Metformin Forest Plot**
